# Tumor microenvironment responsive metal nanoparticles in cancer immunotherapy

**DOI:** 10.3389/fimmu.2023.1237361

**Published:** 2023-07-27

**Authors:** Rou Yang, Lu Chen, Yiling Wang, Lijuan Zhang, Xi Zheng, Yong Yang, Yuxuan Zhu

**Affiliations:** Department of Pharmacy, Personalized Drug Therapy Key Laboratory of Sichuan Province, Sichuan Academy of Medical Science & Sichuan Provincial People’s Hospital, University of Electronic Science and Technology of China, Chengdu, China

**Keywords:** metal nanoparticles, tumor microenvironment, cancer immunotherapy, unique responsive, tumor targeting

## Abstract

Malignant tumors have a unique tumor microenvironment (TME), which includes mild acidity, hypoxia, overexpressed reactive oxygen species (ROS), and high glutathione (GSH) levels, among others. Recently, TME regulation approaches have attracted widespread attention in cancer immunotherapy. Nanoparticles as drug delivery systems have ability to modulate the hydrophilicity of drugs to affect drug uptake and efflux in tumor. Especially, the metal nanoparticles have been extensive applied for tumor immunotherapy due to their unique physical properties and elaborate design. However, the potential deficiencies of metal nanoparticles due to their low biodegradability, toxicity and treatment side effects restrict their clinical application. In this review, we briefly introduce the feature characteristics of the TME and the recent advances in tumor microenvironment responsive metal nanoparticles for tumor immunotherapy. In addition, nanoparticles could be combined with other treatments, such as chemotherapy, radiotherapy and photodynamic therapy also is presented. Finally, the challenges and outlook for improving the antitumor immunotherapy efficiency, side effect and potential risks of metal nanoparticles has been discussed.

## 1 Introduction

Cancer is a general term for a broad group of various diseases that can afflict any area of the body. The alternative terms used are malignant tumors and superfluous organisms. It becomes one of the leading causes of death in the worldwide, and it is reported that nearly 10 million deaths were caused by cancer in 2020, accounting for about one-sixth of global deaths (1). It is therefore vital to promote and forge new approaches to fight against tumors. In an effort to more effectively treat cancer, many researchers are focusing on the cellular and molecular mechanisms of tumors, and the characterization of cancers to allow for the development of new highly effective antitumor drugs.

**Figure 1:**
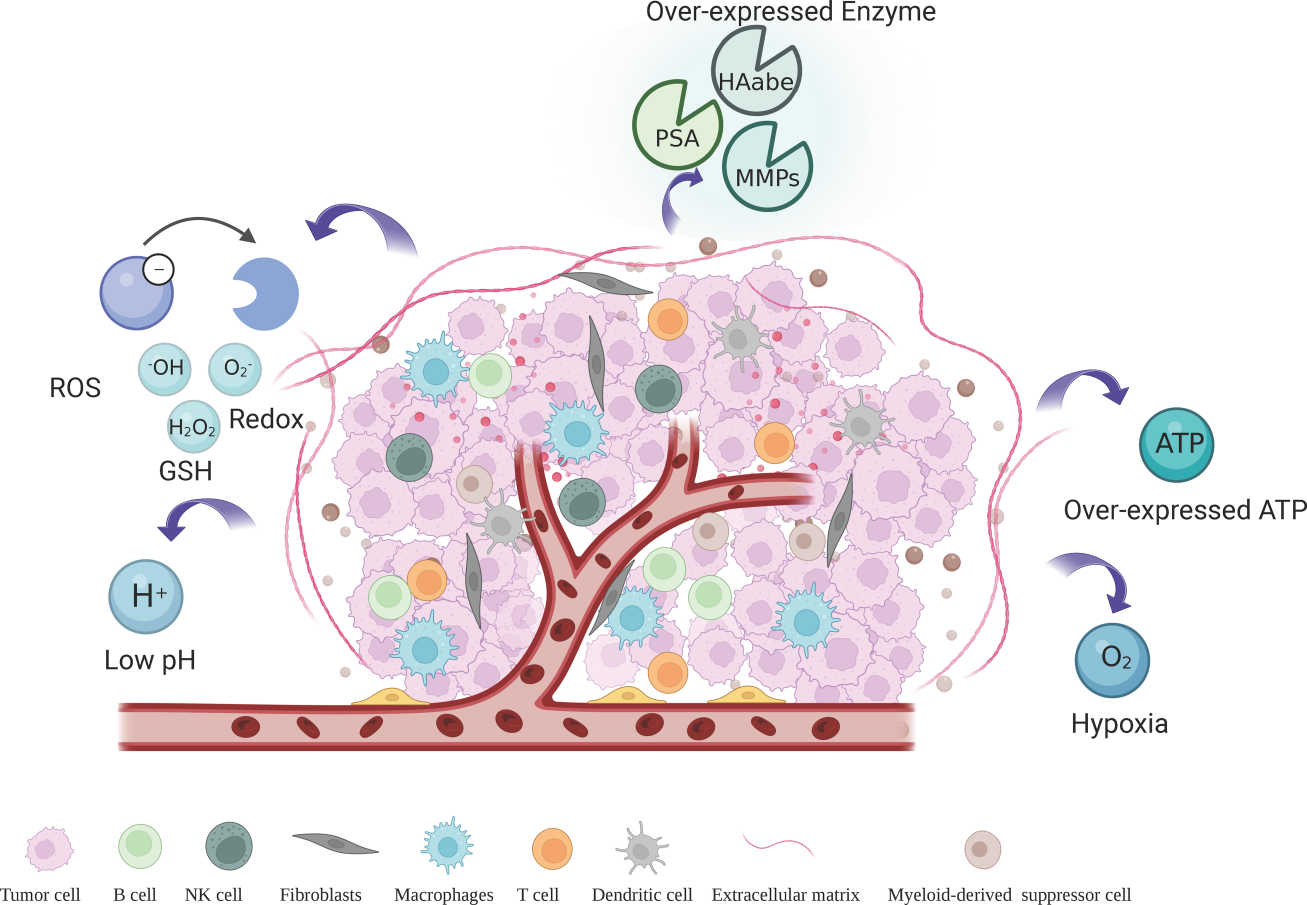
Tumor microenvironment and feature.

The tumor microenvironment (TME) generated by solid tumors is attributed to key parameters that promote tumor growth, proliferation, angiogenesis, invasiveness, and metastasis, which has been considered one of the major obstacles in the cancer treatment process (2). The TME is composed of multiple intertwined cellular and non-cellular components, the major components of which include immune cells, pericytes, tumor-associated endothelial cells (TECs), tumor-associated fibroblasts (CAF), extracellular matrix (ECM), and peripheral blood vessels (**Figure 1**). TME also has a series of unique pathophysiological features including hypoxia, acidic pH, high levels of reduced glutathione (GSH) and reactive oxygen species (ROS), vascular abnormalities, specific metabolism, and overexpression of certain specific enzymes (3). It is possible to design drug delivery systems with targeted and intelligent response release based on the distinctive biological properties of TME.

In recent years, with the profound research of tumor microenvironment, the tumor immunotherapy is becoming a proven and crucial therapy for cancer. Tumor immunotherapy avoids greater damage to normal cells by activating the patient’s own immune system to recognize and eliminate tumors, while improving systemic immunity to inhibit tumor metastasis. However, immunosuppression caused by tumor microenvironment is one of the most difficult obstacles in tumor immunotherapy (4, 5). During antitumor therapy, the tumor microenvironment can increase immunosuppression and tumor cell evasion through a series of mechanisms that induce immune tolerance and suppress antitumor immune responses. It is found that tumor-associated macrophages, fibroblasts, and regulatory T cells in the TME can mediate innate immunity and generate antitumor and pro-tumor responses according to the regulation of signaling pathways and chemokines (6). Furthermore, the high expression of immune checkpoints in TME contributes to tumor immune escape. TME can also regulate drug function and penetration as well as enhance drug resistance by protecting pro-proliferative factors in TME and tumors (7). The complex interactions between tumor cells and TME continue to influence tumor progression, therapeutic efficiency and drug resistance. Consequently, the targeted nano-drug delivery systems could be flexibly designed to reshape the tumor microenvironment to address the therapeutic barriers caused by the immunosuppressive tumor microenvironment, which is hopefully enhancing the effectiveness of tumor immunotherapy.

The TME of solid tumor has distinctive features including low pH, hypoxia and immunosuppressive environments that offer new insights and possibilities for specific cancer therapies. Metal nanoparticles have a variety of unique properties which being considered as the ideal candidate for TME modulation. Metal nanoparticles have been used in various studies of TME modulation, including acidic pH, hypoxia and immunosuppression of TME (8). It is reported that metal nanoparticles could be stimulated by different physical factors to produce highly toxic ROS in the TME. Oxidative stress occurs when ROS levels exceed the antioxidant capacity of cells, ultimately leading to tumor cell death (9). Furthermore, it has been shown that the efficacy of photodynamic therapy and radiotherapy is highly dependent on the level of oxygen in the tumor, moreover the hypoxic microenvironment in the tumor could reduce the effectiveness of the treatment (10, 11). Moreover, the hydrogen peroxide produced by the reaction of metal peroxides with water in an acidic environment not only induces oxidative stress, but also generates additional oxygen (12). The reason is that hydrogen peroxide acts as a reactive substrate for molecules such as catalase, alleviating hypoxia in the tumor microenvironment.

Metal nanoparticles have been developed for more than two decades, which have been a trendy topic in nanocarrier materials research due to their unique properties, suitable and tunable drug delivery functions. Owing to the complex tumor immune escape mechanism, the positive rate of immunotherapy in clinical treatment still needs to be improved. Moreover, immunotherapeutic drugs are often distributed to various tissues and organs throughout the body, which cannot achieve precise targeting to the lesion, and causing a series of immune-related adverse reactions. With the development of metal nanoparticles in synthesis and modification strategies, many metals and metallic elements can be formed into nanostructures that can be utilized in the treatment of cancer. It is reported that metal nanoparticles are capable of preventing sudden drug release and avoiding off-target effects, which gives it many advantages such as improved pharmacokinetic properties and flexible control of drug release (13, 14). Moreover, several studies have shown that metal nanoparticles, modulated by surface ligands or intrinsic properties, can respond to the unique chemical properties of TME by precisely delivering immunomodulatory factors to target tissues or specific immune cells, to achieve an effective and enhanced immune response for enhanced therapeutic efficacy (15–17). Recently, there are a large number of metal nanoparticles have been developed, such as gold, silver, copper, bismuth, manganese, iron, platinum and lead, which can be applied in various modalities of cancer therapy in different forms such as antibodies, solids and coatings (18–21). In this review, the current progress of TME-responsive metal nanoparticles for the field of tumor immunotherapy will be summarized, and the main challenges and directions for the development of this field will be outlined.

## 2 Impact of the tumor microenvironment on tumor

TME is a dynamic and sophisticated environment made up of diverse cellular and non-cellular components. Briefly, the TME represents all components of a solid tumor other than cancer cells. Cells in TME interact with each other to affect the progression of tumors (22). This specific broad range of cellular interactions will determine tumor responsiveness to drugs (23, 24).

There are multiple functionally distinct cells in TME. Endothelial cells in TME offer nutritional support for tumor growth and development and protect tumor cells from the immune system. Immune cells in TME include innate and adaptive immune cells. Immune cells are involved in various immune responses and activities that shift the behavior of the tumor and its response to therapy. Significantly, immune cells in TME can exert both opposite anti-tumor and pro-tumor effects through different activation states and localization. The fibroblasts in TME permit cancer cells to translocate from the primary tumor site into the bloodstream for systemic metastasis. The extracellular matrix further influences cancer cell migration by altering the physical properties, composition and topography of cancer cells.

In addition to multiple malignant cells, TME also contains secreted proteins, blood vessels and non-malignant cells that support and influence tumor growth. The cytokines and growth factors have been secreted by tumor cells or stromal cells in the TME, which might contribute to abnormal tumor growth, angiogenesis, metastasis and drug resistance (25–27). It has been found that vessels within tumors have ability to exhibit altered structural and functional properties and lead to hypoxia and limited nutrient supply. Furthermore, TME hypoxia can alter gene expression in tumor cells, thereby increasing tumor cell survival and resistance to apoptosis induction (28). However, it is well known that the actual composition of the tumor microenvironment and how it affects cancer progression may differ from patient and cancer type. The progression and development of cancer are affected by the TME components and are under the control of the host immune system. Therefore, targeting and manipulating cells and factors in the tumor microenvironment during cancer treatment might help control malignancy and achieve positive health outcomes (3).

## 3 Categories of TME-responsive metallic nanoparticles for immunotherapy

The tumor microenvironment is a main obstacle in immunotherapy, which might be associated with the metastasis, resurgence and drug resistance of malignant tumors. It is commonly known that the cancer progression process is not only influenced by the TME component, but also by the control of the host immune system (3). It is reported that TME remains an essential battlefield between the host immune system and tumors. It has been shown that tumors affect TME by actively recruiting and modulating various cellular phenotypes and functions, which aim to promote immunosuppression and tolerance to tumor-associated antigens (29, 30).

**Figure 2:**
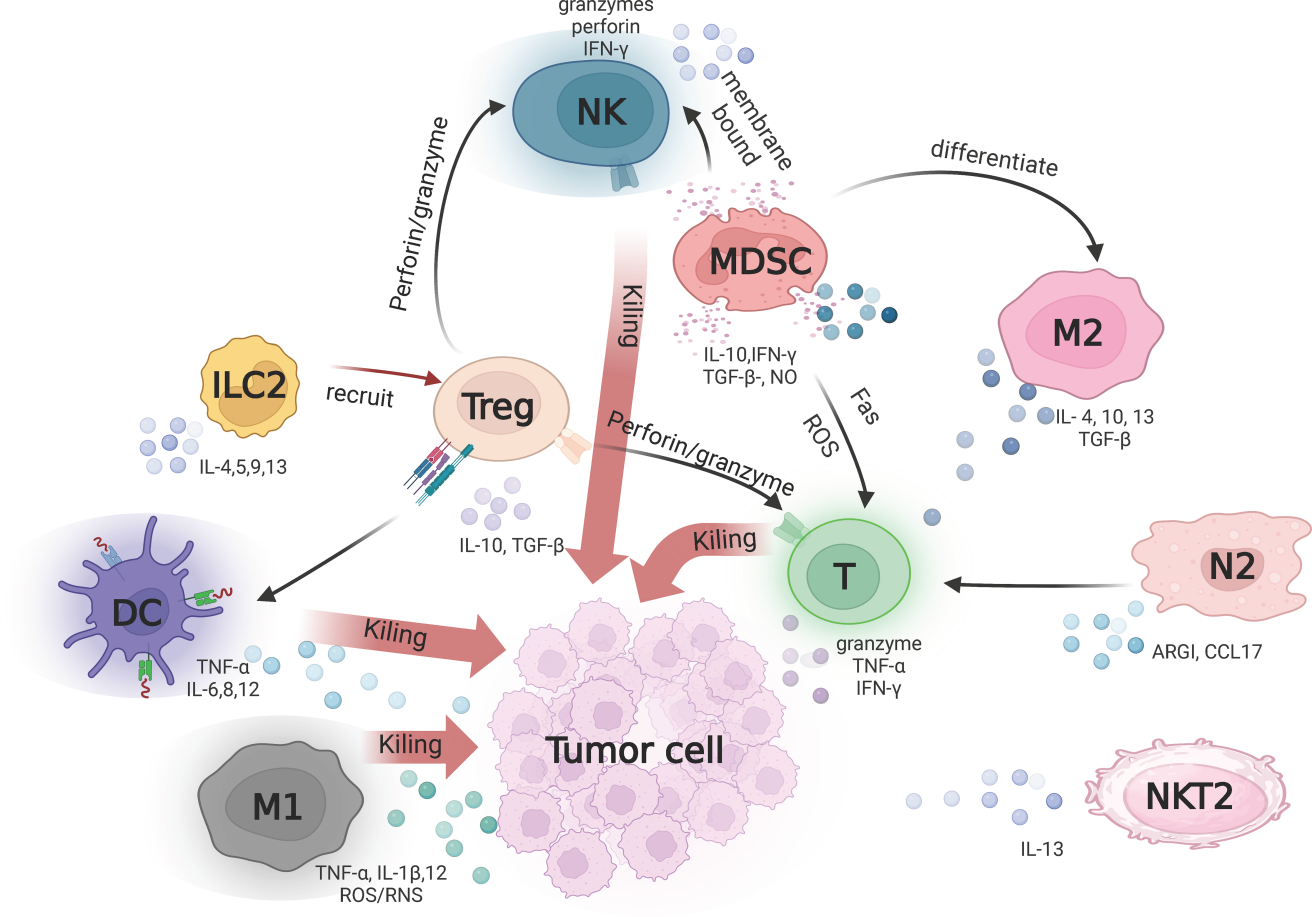
Tumor-associated immune cells in tumor microenvironment. T cells, DCs and M1 contribute to anti-tumor immunity by secreting different signaling molecules such as TNF-α, IL-6 and IL-8 respectively. M1 macrophages exert direct anti-tumor effects through the release of reactive oxygen/nitrogen species (ROS/RNS). Treg, M2, ILC2 and NK2 suppress antitumor immune responses by producing various immunosuppressive cytokines such as TGF-β, IL-10 and IL-13. N2 inhibits T cell proliferation by releasing arginase-1 (ARG1) and solvents the chemokine CCL17 to recruit Tregs.

The first step in regulating TME is to regulate the surrounding cells, mainly including fibroblasts, immune cells and vascular cells (31). One of the mostly significant cell types in the tumor microenvironment is the immune-related cells, including pro-tumor cells and anti-tumor cells. Tumor antagonistic cells associated with antitumor immunity include memory T cells, neutrophils, M1-polarized macrophages, natural killer (NK) cells, and dendritic cells (DC). Tumor-promoting immune cells include regulatory T cells (Treg), M2 phenotype tumor-associated macrophages (TAM), bone marrow-derived suppressor cells (MDSCs), natural killer T Type 2 cells (NKT2), N2-polarized neutrophils, and ILC2s (**Figure 2**) (32–35). The relative numbers of these immune cells and their interrelationships are associated with immunosuppression (36). According to the underlying mechanisms, immunosuppressive pathways can be defined in cytokine-dependent, enzyme-dependent and immune checkpoint-dependent (37, 38). It is found that signaling pathways and chemokines in the tumor microenvironment have ability to regulate immune-related cells to exert anti-tumor and pro-tumor effects. Therefore, tumors can be made more sensitive for immunotherapy by modulating immune signaling pathways, particularly immunosuppressive pathways.

Metal nanoparticles are nanoparticles derived from metal-containing compounds which represent a variety of nanosized materials that carry metal ions and are conjugated to other inorganic or organic molecules (39). These materials are suitable as diagnostic and drug delivery tools for cancer (40). In comparison to other nanoparticles, metal nanoparticles have their unique physical and chemical properties such as magnetic, optical, thermal, catalytic and electrical properties (41). Metal nanoparticles have been widely used in the development of oncology therapeutics due to the exploitation of their properties, which give them the advantage of overcoming biological barriers and alleviating unfavorable counter processing environments (36). Immunotherapy has now emerged as an attractive option for cancer treatment, which uses the body’s own immune system to fight tumors (42). Metal nanoparticles not only have a direct toxic effect on tumor cells by mediating the reorganization of the extracellular matrix, but also have ability to activate the immune response. Metal nanoparticles interact with Toll-like receptors in order to initiate pro-inflammatory polarization of macrophages. Subsequently, T cells can be activated by macrophages or metal nanoparticles to achieve anti-tumor cytotoxicity (39). Additionally, combining metal nanoparticles with immunotherapy endows unique advantages among numerous immunotherapy pathways, as metal nanoparticles can obtain safer and more effective cancer treatment by providing targeted distribution of immune drugs and greatly enhancing drug bioavailability (43, 44). Because of the physiological barriers, there is a great need for more advanced nanomedicine systems that can effectively and intelligently target TME to enhance treatment outcomes. Metal nanoparticles like magnetic nanoassemblies, gold-based nanoassemblies, calcium-based nanoassemblies, mesoporous nanoassemblies, metal-organic frameworks (MOFs) and manganese-based nanoassemblies have been exploited for improved cancer immunotherapy. At the very same time, the efficacy of a single therapeutic modality is often limited given the tumor suppressive effect. There is also a considerable amount of research into the combination of metal nanoparticles with various types of therapeutic modalities, such as chemodynamic therapy (CDT), photodynamic therapy (PDT), photothermal therapy (PTT), radiation therapy (RT) and immunotherapy. Many metals and metal elements have unique properties that allow precise pairing with other molecules, such as photosensitizers, chemotherapeutic agents and enzymes, ultimately enabling combination therapies including immunotherapy, photodynamic therapy and chemotherapy (45, 46). Metal-based nanoparticles have been demonstrated to reach superior anti-antitumor effects in combination with other therapies (47–49). For enhancing anti-tumor immunotherapy, various metal nanosystems have been reported as shown in **Table 1**.

**Table 1:** The Summary of metal nanoparticles modulating TME to enhance tumor immunotherapy.

Responsive	Materials	Immunotherapeutic drug	Application	Reference
pH	AuNBP@CuS		Photothermal therapy, photodynamic therapy, chemodynamic therapy, immunotherapy	(48)
	Gd-MOF-5	αPD-L1	Immunotherapy	(50)
	Fe-MOF		Immunotherapy	(51)
	MnO@mSiO_2_-iRGD	αPD-L1	Chemodynamic therapy, immunotherapy	(52)
	copper-iron peroxide nanoparticles		Chemodynamic therap	(53)
	PFG MPN	PD-L1	Photothermal therapy, immunotherapy	(54)
	DOX-TAF@FN	DOX	Chemodynamic therapy, immune therapy	(55)
GSH	Cu-MOF	IDO	Immunotherapy	(56)
	miDAC@PDA	DOX	Chemodynamic therapy, immunotherapy, Photothermal therapy	(57)
	Au@MnSe_2_		Chemodynamic therapy, photocatalytic therapy, photothermal therapy	(58)
	TfRA-AuNCs		Immunotherapy	(59)
	Fe_3_O_4_ nanoparticles		Immunotherapy	(60)
	Fe-PHCN@DOX	DOX	Immunotherapy	(61)
	HA@Cy-Cu NPs		Chemodynamic therapy, photothermal therapy	(47)
ROS	Fe_3_O_4_ nanoparticles	αPD-L1	Chemodynamic therapy, Immunotherapy, Photodynamic therapy	(62)
	Cu@COF-TATB		Immunotherapy	(63)
	zGd-NRs		Radioimmunotherapy	(64)
	S-AuNC	αPD-L1	Immunotherapy, radiotherapy	(65)
	COFTFBE-PDAN@FeIIITA-PEI		Ferroptosis therapy	(66)
	MnO_2_@TPP-PEG		Photodynamic therapy, chemodynamic therapy	(49)
	Cu_2_-xSe nanoparticles	αPD-L1	Immunotherapy	(67)
	Cu/CaCO_3_ @Ce_6_, CCC		Chemodynamic therapy, sonodynamic therapy	(68)
Hypoxia	manganese ferrite nanoparticles	DOX	Chemoimmunotherapy	(69)
	CeO_2_ nanoparticle		Photodynamic therapy, photothermal therapy	(70)
	TiO_2_@Pt		Sonodynamic therapy, starvation therapy	(71)
enzyme	SPIO NP@M-P	PD-L1	Immunotherapy	(72)
	CM@Mn	PD-L1	Immunotherapy	(73)
	PL/APMP-DOX	DOX	Immunotherapy	(74)
ATP	IR@ZIF-RGD		Immunotherapy	(75)
	MnFe_2_O_4_-DCA		Immunotherapy	(76)
pH+GSH	PC/iron ions/TA		Ferroptosis therapy, photothermal therapy	(77)
	DOX@MSN@CuO_2_	DOX	Chemodynamic Therapy, Chemotherapy	(78)
	NLG919/PGA-Cys-PPA@Gd	IDO inhibitor	photodynamic therapy, Immunotherapy	(79)
pH +ROS	Fe^2+^@UCM-BBD	DOX	chemodynamic therapy, photodynamic therapy	(80)
pH+Hypoxia	Metal-phenolic networks	LOX、ATO	sonodynamic therapy	(81)

### 3.1 pH-responsive

Numerous studies have shown that the tumor microenvironment is weakly acidic with a pH of approximately 6.5 to 6.8 due to inadequate perfusion, accumulation of lactate, and dysregulated energy metabolism. It has been shown that the acidic tumor microenvironment is essential for the development of malignancy and the evolution of somatic cells. Furthermore, the acidic tumor microenvironment can influence the malignant behavior of tumors, including metastasis and invasion rates, and affect the mechanisms of immune surveillance (82). Therefore, the pH of the tumor microenvironment appears to be an essential therapeutic objective in the administration of cancer. Given the pH difference between normal tissue and tumor microenvironment, pH-responsive metal nanoparticles are now commonly designed for cancer therapy. Metal nanoparticles can be used as drug carriers for antitumor drug delivery, degrading based on the acidic tumor microenvironment environment, liberating metal ions and antitumor drugs to inhibit or kill tumor cells through combined photothermal therapy, chemotherapy and immunotherapy.

One of the fundamental properties of metallic nanoparticles, particularly nanoparticles such as gold, silver and copper, is the presence of surface plasmon resonance (SPR). SPR is a phenomenon in which photons cause the oscillation of free electron on the metal surface when light is incident on the metal-dielectric interface (83). The SPR will be more pronounced in nanoparticles because of their higher surface to volume ratio in comparison to their bulk counterparts. SPR properties have been broadly used for photodynamic therapy *via* nanoparticles. In particular, metal nanoparticles can be structured to react to near-infrared absorption to obtain photothermal agents to therapeutic tumors (84). Chen et al. incorporated gold nanobipyramids and copper sulfide in a core/shell structure to form pH-responsive nanoparticles AuNBP@CuS (48). In the acidic tumor microenvironment, Cu^2+^ released from CuS can be converted to Cu^+^ by glutathione after a Fenton-like reaction with hydrogen peroxide, producing a highly toxic hydroxyl radical in the tumor area. Meanwhile, AuNBP@CuS shows photothermal properties and photodynamic under NIR-II laser irradiation. Damage-associated molecular model molecules invoked by NIR-II photo/chemotherapy in dying cells can concurrently trigger adaptive immune responses. Damage-associated molecular pattern molecules include adenosine triphosphate, preapoptotic calmodulin, and high-mobility group box-1. This report reveals that AuNBP@CuS performs well in tumor suppressors with an effective immune cell death process.

A proactive approach to invigorate the tumor immune microenvironment is to induce immunogenic cell death (ICD) in tumor cells. ICD promotes dendritic cell maturation and infiltration of cytotoxic T lymphocytes by releasing danger-associated molecular patterns (DAMP) and tumor-associated antigens. This procedure may then reverse the tumor immunosuppressive microenvironment to maximize the sensitivity of immunotherapy (85, 86). It is reported that the tumor cell death could be induced by various therapeutic modalities such as radiotherapy and photothermal therapy in an immunogenic manner (87). Metallic nanomaterials offer the potential to deliver optimal doses of ICD inducers to target tissues, which can enhance the effects while reducing side effects. Dai et al. incorporated Gd^3+^ and Zn^2+^ in bimetallic MOF nanoparticles (Gd-MOF-5), which were used as immunomodulators and ICD inducers to modulate immunostimulatory signals (50). It was shown that MOF-5-mediated intracellular Zn^2+^ overload has a strong ability to activate endoplasmic reticulum stress to induce ICD, which greatly facilitates tumor immunotherapy. Gd-MOF-5 combined with anti-programmed death ligand 1 antibody (αPD-L1) triggered a robust immune response and significantly reduced tumor growth.

Tumor-associated macrophages are an integral part of the TME and perform a crucial function in cancer immunotherapy. Macrophages have different phenotypic and metabolic characteristics in response to various stimuli. TAMs mainly present an anti-inflammatory M2 phenotype stimulated by rich anti-inflammatory stimuli that facilitate tumor growth (88). It has been shown that there is multiple M2-associated signaling cues in TME that reduce the potency of M1-type macrophage polarization (89). Furthermore, it is found that certain metallic nanoparticles currently developed can modulate TAMs from pro-tumor M2 to anti-tumor M1-like phenotypes (90). The utilization of metal nanoparticles to remodel TAMs is a prospering modality in cancer immunotherapy. Zheng et al. developed an Fe-MOF nanoparticle loaded with insoluble RSL3 that can degrade and release Fe^3+^ in acidic TME (51). The Fentonian catalytic properties of Fe^3+^ promote lipid peroxidation and disrupt mitochondrial membrane structure, potentially leading to mitochondrial dysfunction. RSL3 is a hydrophobic molecular iron activator that enhances iron-dependent lipid peroxidation in cancer cells and sensitized macrophages. Fe-MOF acts synergistically with RSL3 by boosting iron death-related stress in macrophages that triggers potent pro-inflammatory signaling activation and metabolic conversion, which markedly boosted tumor killing.

These studies show that metal nanoparticles can not only be employed as carriers of drugs for targeted release in the tumor microenvironment to induce immune responses to kill tumor cells, but also directly act as immunostimulatory signal inducers and immunomodulators to trigger immune responses to kill tumor cells, providing further applications of metal nanomaterials in tumor immunotherapy.

### 3.2 GSH responsive

**Figure 3:**
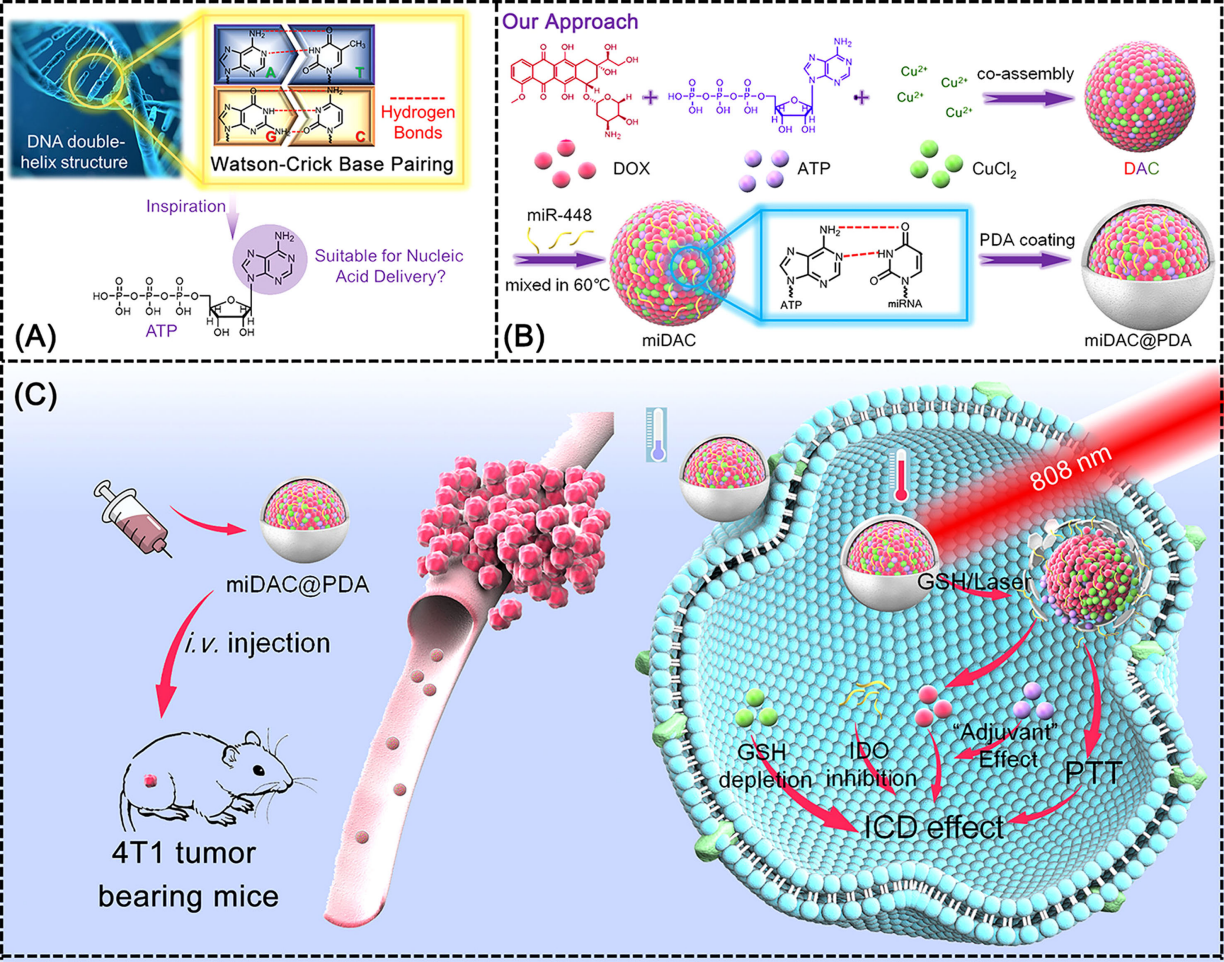
Schematic illustration of miDAC@PDA preparation and its potential mechanism for cancer immunotherapy (57). **(A)** Inspiration from the DNA double-helix structure and the potential nucleic acid delivery capability of ATP; **(B)** preparation pathway of miDAC@PDA; **(C)** potential therapeutic mechanism of miDAC@PDA for synergistic ICD induction.

One of the richest reducing cellular metabolites is GSH, which plays an essential function in maintaining the redox balance in cells (91). In Addition, GSH is involved in regulating protein folding by mediating the generation and degradation of disulfide bonds in many proteins. Because of abnormal metabolism of tumor tissues, the concentrations of reducing substances in tumor are far higher than those in normal cells (92, 93). Consequently, GSH has been applied as a specific marker for the design and construction of metal nanocarrier materials targeting TME of malignant tumors. Du et al. utilized GSH-sensitive Cu-MOF packed with an inhibitor of the immunosuppressive enzyme IDO and a nitric oxide (NO) donor-nitrosothiol moiety to construct a nanomedicine drug (BMS-SNAP-MOF) (56). High levels of GSH in TME trigger a cascade reaction with Cu-MOF, releasing IDO inhibitors and producing plentiful NO in situ. IDO inhibitors and NO synergistically modulate the immunosuppressive tumor microenvironment, increasing CD8+ T cells and decreasing Treg cells, thereby reducing immune escape and enhancing immunotherapeutic efficacy. Biocompatible, safe and physiologically stable Cu-MOF displays significant future potentials as a drug carrier. Furthermore, metal-based nanoparticles can be used in combination with chemical drugs, physical therapy and photothermal therapy to co-induce ICD. Jiulong et al. co-assembled adriamycin (DOX), adenosine triphosphate and copper ions into ligand polymer nanoparticles, afterwards combined microRNA (miRNA) then further coated with polydopamine (PDA) finally form the miDAC@PDA (**Figure 3**) (57). The prepared miDAC@PDA nanoparticles can efficaciously accumulate into tumor tissues to liberate drugs in response to laser irradiation and high levels of GSH. Cu^2+^ mediated GSH depletion disrupts tumor redox homeostasis, thereby amplifying the immunogenic cell death cascade response. This composite metal nanoparticle also activates cytotoxic T-cell infiltration and reshapes the tumor immunosuppressive microenvironment, combining with chemotherapy and photothermal therapy for immunotherapy of tumors.

In besides, GSH is an antioxidant of endogenous origin that protects cells from damage caused by various oxidants such as lipid peroxides and reactive oxygen species. High concentration of GSH in TME is strongly linked to tumor progression and increased resistance to antitumor drugs, which can diminish the efficacy of antitumor therapy by depleting ROS generated in multiple antitumor therapy types such as CDT and PDT. Therefore, down-regulation of over-expressed GSH in tumor might be an attractive tactic that could improve the effectiveness of tumor therapy. Wang et al. synthesized a core-shell form of gold-manganese selenide nanoparticles (Au@MnSe_2_ NPs) of which MnSe_2_ depletes GSH in TME by converting it to Mn^2+^ and the presence of Au further catalyzes the reaction (58). Au@MnSe_2_ NPs have robust photothermal conversion efficiencies and stabilization under NIR light irradiation, transforming the absorbed NIR light into heat for tumor eradication and accelerating the CDT and PCT effects. Au@MnSe_2_ NPs also activated cytotoxic and accessory T lymphocytes and augmented the anti-tumor efficacy of T cells.

Ferroptosis is a novel non-apoptotic programmed cell death that is strongly correlated with tumor progression (94). Ferroptosis is the result of cellular metabolism and dysregulation of redox homeostasis induced by lethal lipid peroxidation, which may be suppressed by directly blocking lipid peroxidation or by depleting iron through genetic means or pharmacological. Significantly, there is growing evidence for a potential physiological effect of Ferroptosis in tumor immunity and suppression (95). GSH consumption has been shown to be effective in enhancing ferroptosis. Dan Zhao et al. constructed fluorescent gold nanoclusters (TfRA-AuNCs), which induced efficient hydroxyl radical generation and GSH depletion, and inhibited tumor growth through enhanced ferroptosis effect (59). Meanwhile, Fe_3_O_4_ nanoparticles synthesized by Cong Wu et al. have the ability to induce ferroptosis in macrophages (60). They observed that Fe_3_O_4_ nanoparticles were capable of decreasing the glutathione/oxidized glutathione (GSH/GSSG) ratio and suppressing the oxidative stress state in tumor cells, reducing the macrophage viability, and inducing macrophage polarization toward the M1 phenotype. The present study demonstrates the enormous possibility of metal nanoparticles for Ferroptosis treatment in reversing drug resistance to cancer therapy.

### 3.3 ROS responsive

It has recently been demonstrated that the increased metabolism due to abnormal cancer cell outgrowth leaves cancer cells in a continuous state of oxidative stress, contributing to elevated basal levels of ROS in TME. ROS plays a complicated and multi-faceted role in TME, facilitating tumor progression and metastasis by a multitude of mechanisms such as the promotion of cellular transformation and tumor angiogenesis, and can therefore be used to create ROS-responsive nanoparticles for cancer immunotherapy (96). Pre-drugs that are specifically activated by ROS in tumor cells can be medically enhanced for drug specificity to tumor cells. Tumor development can be suppressed by delivering and liberating therapeutic drugs such as αPD-L1 and chemotherapeutic agents into tumor cells by combining metal nanoparticles with ROS-responsive prodrugs. Ding et al. developed a ROS-responsive Fe_3_O_4_ nanoparticle that was cross-linked to αPD-L1 prodrug nanoparticles *via* a ROS-responsive linker (62). Fe_3_O_4_ nanoparticles can produce toxic hydroxyl radicals (**·**OH) in the tumor microenvironment for CDT by the Fenton reaction. The cumulative effect of CDT leads to substantial ROS formation to ablate tumor cells, while inducing ICD and the liberation of αPD-L1 out of pre-drug nanoparticles. The combination of ICD and αPD-L1-mediated immune checkpoint blockade therapeutics induces the initiation of effective T cells by boosting the maturation of DCs and decreases the number of regulatory T cells in the tumor, triggering a powerful anti-tumor immune effect that demonstrates a favorable inhibitory effect.

**Figure 4:**
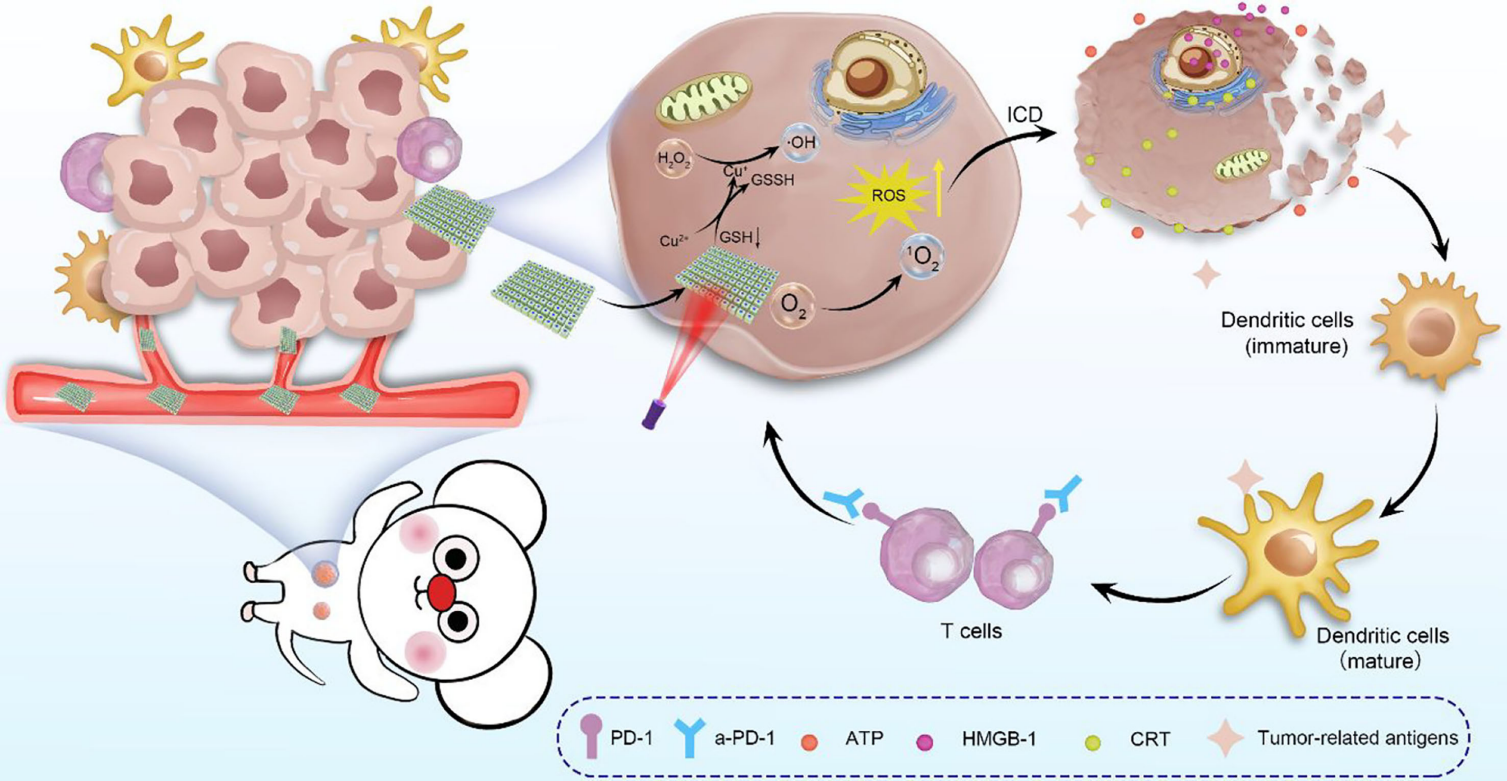
Schematic illustration of 3D Cu@COF-TATB for cancer immunotherapy *via* PDT- and CDT- triggered ICD (63).

Oxidative stress occurs when ROS levels overwhelm the antioxidant capacity of cells, leading to cell death. Oxidative stress also affects immune cells in TME, where ROS is one of the essential signaling mediators involved in the activation of T cells and NK cells. Slight elevation of ROS induces T cell activation and differentiation, but ROS can also result in the death of activated T cells and maintenance of immune homeostasis. It is found that ROS has been applied by neutrophils and macrophages to destroy cancer cells. Increased ROS in TME is a feature of chronic inflammation, and the interplay between inflammation and oxidative stress mediators can impair the immune response to tumors (97). As a result, ROS in TME and other mechanisms combine to form immune tolerance to tumors. By delicately managing ROS levels in TME, the detrimental effects of ROS can be avoided to enhance antitumor efficacy. Metal nanoparticles are outstanding in terms of oxidative stress induction in cells, and hence many metal nanoparticles have been widely used in recent years to enhance tumor immunotherapy efficacy by modulating ROS levels in TME. Zhang et al. embedded copper ions (Cu^2+^) in a three-dimensional covalent organic framework to obtain multifunctional metal nanoparticles 3D Cu@COF-TATB (**Figure 4**) (63). Cu^2+^ can catalyzed the production of hydroxyl radicals (**·**OH) *via* the Fenton reaction, while different types of ROS are efficiently produced to enhance the anti-tumor therapeutic effect. The resulted ROS can be further induced by ICD of cancer cells to boost immunogenicity and further activate the body’s anti-tumor immune response.

Radiotherapy has been demonstrated to be one of the most efficient local therapies for tumors. Radiation can ionize cellular DNA directly or generates a variety of ROS to liberate tumor-associated antigens and drives the secretory of damage-associated molecular patterns (DAMP) within the TME. Therefore, radiation can in practice theoretically induce ICD. but the immunogenic properties of tumor cells are apparently quite poor, and radiation alone can only induce a low level of ICD. Consequently, tumor targeted by metal nanoparticles piggybacking on drugs can be achieved to promote immune activation and enhance checkpoint blockade immunotherapy against sexual tumors. Huang et al. have constructed a nanoparticle (ZGd-NRs) based on gadolinium and zolayic acid (64). ZGd-NRs can be used to induce ICD by depositing X-rays, enhancing ROS production and accumulation, while consuming tumor-associated macrophages and inhibiting regulatory cytokines. This metal nanoparticle promotes antigen presentation, immune initiation and T-cell infiltration through a synergistic strategy of ICD induction and modulation of the immunosuppressive microenvironment, and greatly enhances the efficacy of tumor immunotherapy.

It was suggested that the different levels of ROS in TME have two different effects of supporting and inhibiting tumor growth. The accumulation of ROS in tumor cells has ability to be not only metabolic by-products but also positively contributes to the therapeutic effect. Hence the combined application of modulating ROS levels in TME by metal nanoparticles with immunotherapy is becoming a hopeful therapeutic strategy.

### 3.4 Hypoxia responsive

Hypoxia is an essential characteristic commonly found in many solid tumors and is mainly caused by an imbalance between the oxygen supply and oxygen consumption of the tumor tissue (98). Hypoxia could lead to immunosuppression, primarily by upregulating the expression of certain chemokines and the build-up of bone marrow-derived suppressor cells and regulatory T cells (99, 100). Hypoxia further promotes the transformation of macrophages and neutrophils into a tumor-promoting M2 phenotype and inhibits the killing effect of T cells and NK cells, giving tumor cells an immune escape potential. The hypoxic tumor microenvironment limits the antitumor immune response and drives the immune system toward accelerated tumor growth, resulting in further tumor deterioration. Yang et al. developed a hypoxia-sensitive metal nanoparticles, manganese ferrite nanoparticles (MFNs), while encapsulated with adriamycin (Dox), for surmounting tumor immune tolerance due to hypoxia as a way to promote chemoimmunotherapy of tumor (69). The DOX-loaded metal nanoparticles exhibited significant inhibition of primary tumor growth. Consequently, the modulation of hypoxic tumor microenvironment contributes to a durable immune memory effect to avoid tumor recurrence and metastasis.

**Figure 5:**
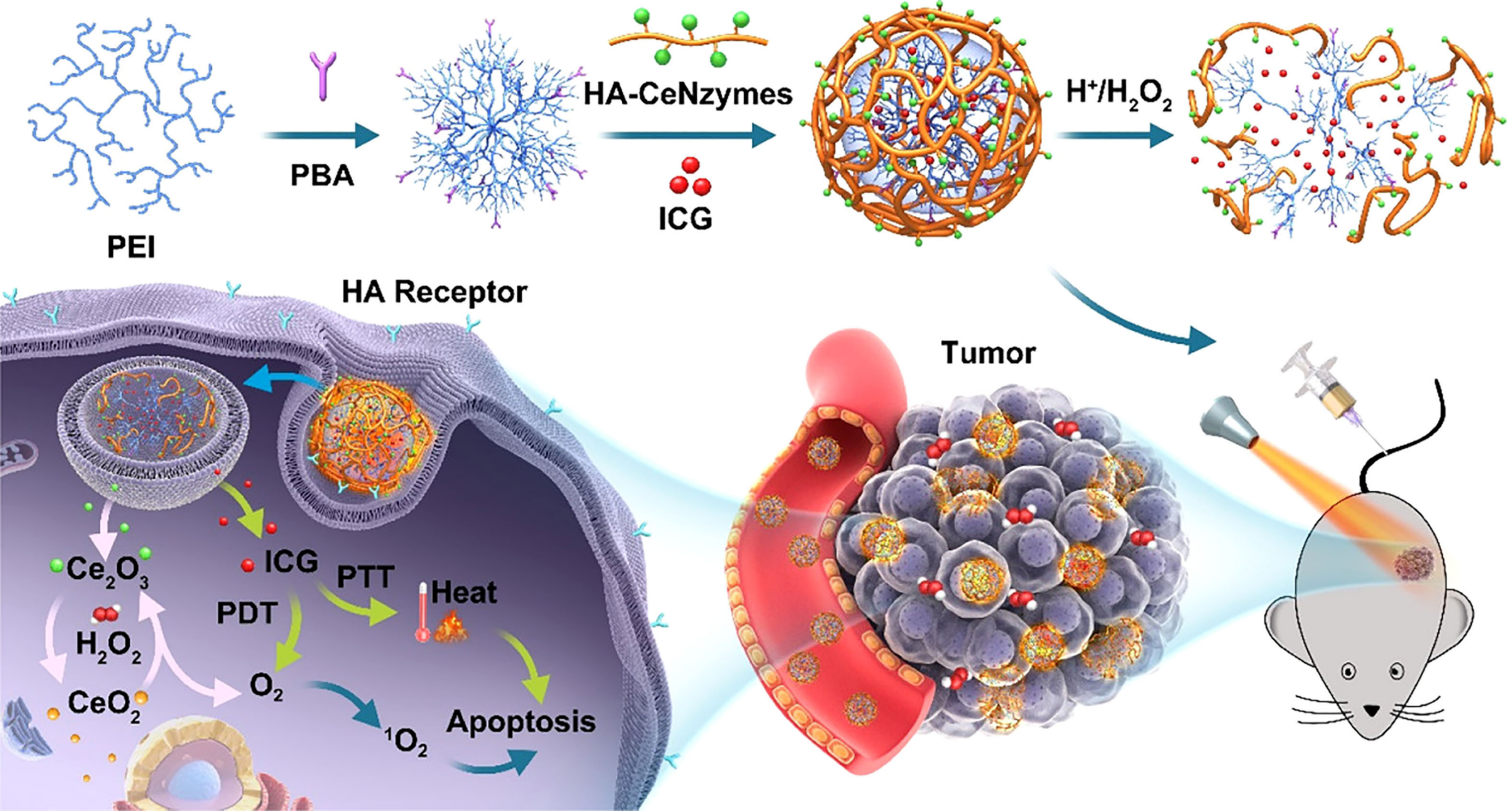
Illustration of *in vivo* regenerable cerium oxide nanozyme-loaded pH/H_2_O_2_-responsive nanovesicle for tumor-targeted PDT (70).

Hypoxia has an incredibly significant impact on tumor vascularity, growth, and metastasis, and also directly as well as indirectly impairs therapeutic efficacy or leads to increased genetic instability, making it more likely that tumors will acquire drug resistance, and has been considered one of the main obstacles in the process of tumor treatment. Furthermore, hypoxia is among the causes of therapeutic resistance, particularly for PDT and radiotherapy, where oxygen molecules are essential for the eradication of tumor cells. Metal oxides can show functions similar to catalase (CAT) and superoxide dismutase and are commonly used to design nano-delivery systems for tumor hypoxic microenvironment environments. Zeng et al. developed a cerium oxide (CeO_2_) nanoparticle synergistic photosensitizer for improving the tumor hypoxic microenvironment to optimize the efficacy of PDT and PTT (**Figure 5**) (70). Cerium oxide nanoparticles were selectively enriched in TME and generated oxygen through the action of catalase, which significantly improved the hypoxic microenvironment in tumors. Zhao et al. developed a TiO_2_ and Pt-based metal nanoparticle TiO_2_@Pt/GOx (TPG) to mediate starvation therapy (ST) and sonodynamic therapy (SDT) to alleviate TME hypoxia and improve tumor immunosuppression, abnormal angiogenesis and lymphatic angiogenesis (71). TiO_2_@Pt has ability to promote the monomeric oxygen and hydroxyl radical generation, resulting in the accumulation of ROS. Therefore, the combination therapy of ST and SDT triggers strong immunogenetic cell death and produces inhibition of tumor growth and metastasis.

### 3.5 Enzyme responsive

It has been suggested that enzymes in the tumor microenvironment exhibit distinct expression levels from normal tissues. TME displays excessive enzyme secretion, including esterase, hyaluronidases, proteases and γ-glutamyl transpeptidase (101). In particular, proteases play a vital role in catalyzing reaction that irreversibly degrade proteins and maintain the stability of the internal tumor environment (102). Abnormal hydrolysis of proteases in tumors will induce tissue remodeling, inflammation and activation of tumor growth factors, contributing to tumor growth and metastasis. The main proteases in TME include metalloproteinases, troponin and trypsin. Metalloproteinases (MMP) are of prominent importance among the proteases. MMP substantially affects tissue integrity, immune cell aggregation and tissue turnover by degrading extracellular matrix components and selectively releasing cell surface bound cytokines, growth factors or their receptors (103). The homeostasis of protein hydrolysis mediated by metalloproteinases actively promotes the generation of the tumor microenvironment. Accordingly, metal-nanoparticles were designed to target the proteases in TME for enhancing immunotherapy of cancer. Meng et al. developed a multifunctional delivery system based on iron oxide nanoparticles (72). It is discovered that iron oxide nanoparticles (SPIO NP@M) can be encapsulated in lung cancer cell membranes, which has been coupled with PD-L1 inhibitory peptide (TPP1) and MMP2 substrate peptide (PLGLLG). Through the specific action of both TPP1 and PLGLLG peptides, SPIO NP@M was delivered and released into the tumor microenvironment narratively. SPIO NP@M could effectively prolong the half-life of TPP1 peptide and has the ability to reactivate T cells and suppresses tumor development, exhibiting an array of merits including low toxicity, directed release and prolonged *in vivo* circulation.

**Figure 6:**
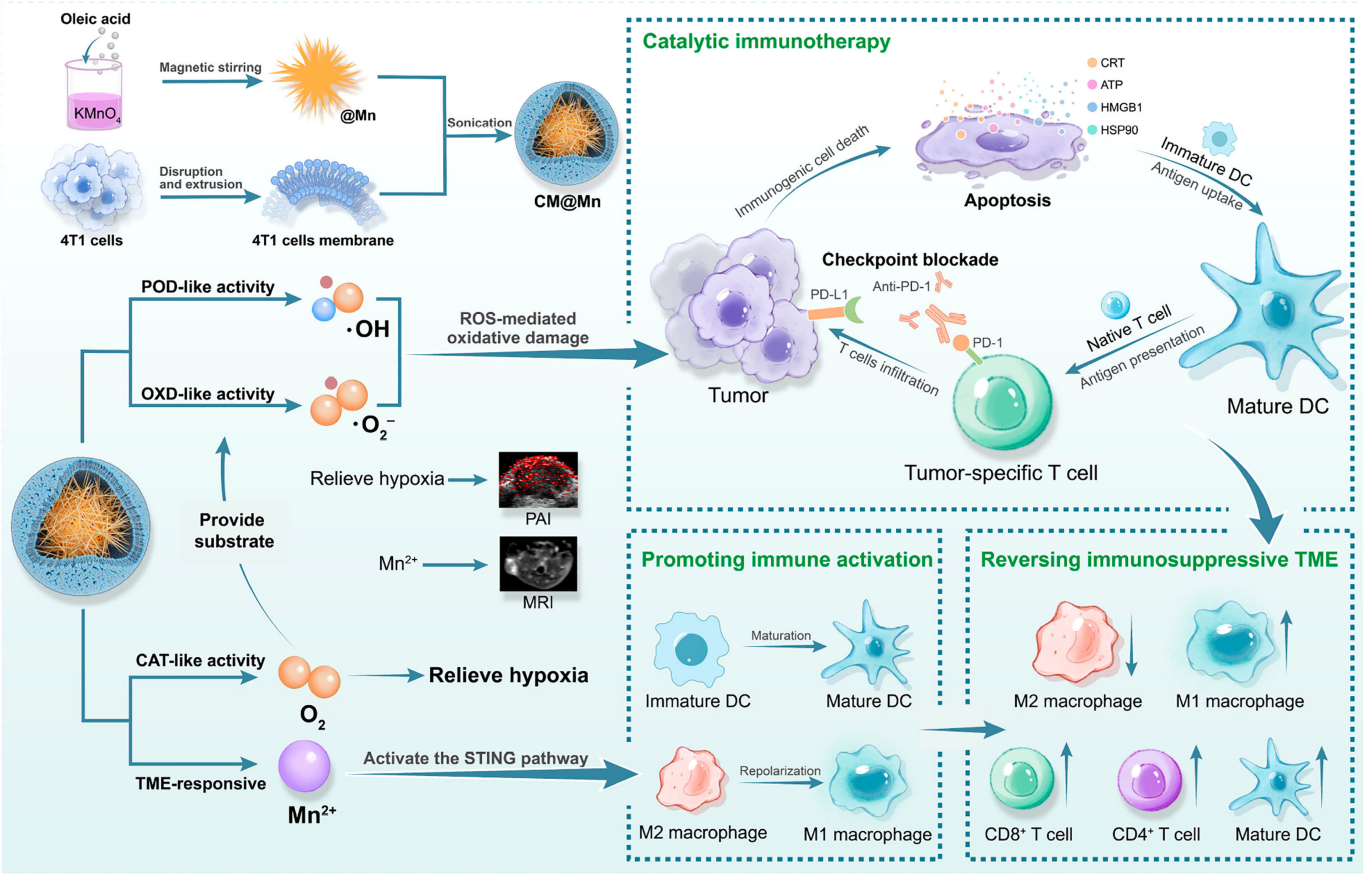
Schematic illustration of the process of preparation of CM@Mn nanozyme and the therapeutic strategy of TME-activable manganese-boosted catalytic immunotherapy combined with PD-1 checkpoint blockade (73).

The cyclic GMP-AMP synthase (cGAS)/interferon gene stimulator (STING) pathway, a key part of innate immunity, has lately been exploited as a progressive candidate for augmenting tumor therapy. Extensive studies have shown that Mn^2+^ can boost the activation of cGAS/STING to stimulate dendritic cell maturation and macrophage M1 polarization, ameliorate immunosuppression, and enhance the efficacy of tumor immunotherapy. Mn-based metal nanoparticles have been studied and synthesized in combination with DOX or PD-1 checkpoint blockade to synergistically enhance cancer immunotherapy. Zhao et al. encapsulated multi-enzyme-mimetic manganese oxide (MnOx) and αPD-1 with tumor cell membranes (CM) to form the nanoenzyme CM@Mn (**Figure 6**) (73). This nanoenzyme exhibited intrinsic peroxidase and oxidase-like activity in TME, generating toxic hydroxyl and superoxide radicals that can cause ICD and kill tumor cells. Furthermore, Mn^2+^ release in TME directly promotes dendritic cell maturation and macrophage M1 repolarization, leading to the reversal of immunosuppressive TME to an immune activating environment. Mn^2+^ in concert with PD-1 checkpoint blockers presents a potent tumor-specific T cell-mediated antitumor response, inhibits tumor proliferation and metastasis, and induces long-term immune memory effects. Lin et al. constructed hybrid nanoparticles (PL/APMP-DOX NPs) based on manganese phosphate nanoparticles loaded with DOX and phospholipids (74). PL/APMP-DOX NPs triggered the release of DOX in TME to induce DNA damage, and Mn^2+^ enhanced cGAS/STING activity and enhanced immune activation, presenting excellent antitumor efficacy.

### 3.6 ATP responsive

As an essential substance for providing energy to tumors, ATP plays a vital role in the pathogenesis of many tumors and in the interaction between host and tumor. It has meanwhile been indicated that the level of ATP is also correlated with tumor responsiveness to therapeutic agents (104). Due to the unusually high rapid accretion of cancer cells leading to accelerated metabolism and excessive glycolysis, the content of ATP in TME is eventually increased (105). Accordingly, ATP in TME has appeared as an up and coming target for tumor immunotherapy in the context of developing metal nanoparticles. Yu et al. developed a Zn^2+^-based composite nanoparticle (IR@ZIF-RGD), which was capable of releasing drugs to augment ATP hydrolysis and depletion based on high level of ATP-responsive degradation in TME (75). The ATP-responsive nanoparticles contributed to tumor metabolic disorders and reversed the immunosuppressive microenvironment by decreasing intracellular ATP level in tumor cells. IR@ZIF-RGD was confirmed to inhibit the growth and metastasis of primary tumors.

The accumulation of adenosine (ADO) and lactate may not only weaken the functionality of cytotoxic T cells, the maturation of dendritic cells may also be decreases, ultimately resulting in the appearance of an immunosuppressive microenvironment. Lactate in tumors is produced by glycolysis, while ADO is produced by ATP catabolism. The metabolic processes of glucose and ATP in tumors can be regulated by synthetic nanoparticles to alter the levels of lactate and ADO in TME, so as to boost the effectiveness of tumor immunotherapy. Dai et al. developed a nanoparticle (MnFe_2_O_4_-DCA), which can be efficiently accessed into mitochondria (76). MnFe_2_O_4_-DCA downregulates both glucose enzymatic and ATP catabolic processes by affecting immunosuppressive adenosine and lactate levels in TME. ATP responsive nanoparticles have therefore become a topical area in tumor immunotherapy, which may attract more concerns in the near future.

### 3.7 Multiple responsive

Given that cancer is a highly sophisticated disease, it is competent to evolve multiple immunosuppressive and drug resistance mechanisms during tumor progression. For this reason, combining various biological characteristics of TME such as pH, ROS, and GSH, the exploitation of multi-responsive metal nanoparticles presenting prominent anti-tumor advantages has become a trend of current research.

#### 3.7.1 pH/GSH

**Figure 7:**
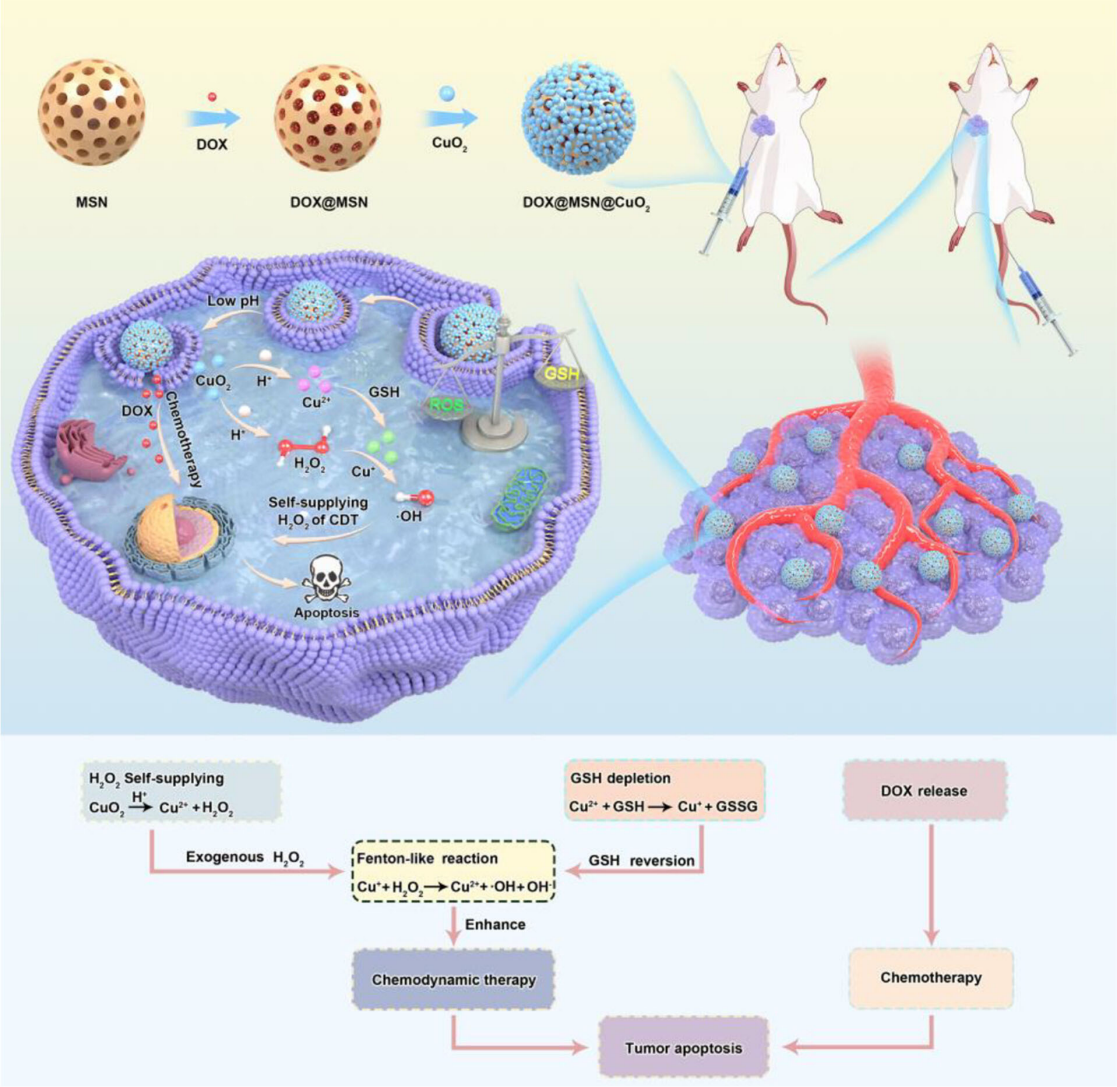
Schematic of DOX@MSN@CuO_2_ synthesis and H_2_O_2_ self-supply and the mechanism of synergistic therapy against tumor cells (78).

Low pH and high levels of GSH are two prominent characteristics of TME that may be used to collaboratively control drug release in TEM. Meng et al. constructed Versatility pH/GSH-responsive and Fenton-reactive nanohybrids of polycysteine/iron ions/tannic acid (PCFT) (77). PCFT releases high concentrations of iron ions into tumor cells in TME based on pH and GSH response, and iron ions catalytic endogenous H_2_O_2_ from tumor cells into highly toxic hydroxyl groups *via* the Fenton reaction to induce ferroptosis therapy (FT). FT kills cancer cells by membrane accumulation of lipid peroxides and downregulation of glutathione peroxidase-4. Several studies shown that tumor cells can be obliterated by PCFT and exhibit outstanding antitumor efficacy. Additionally, Tang et al. developed a pH-responsive and GSH-depleted metal nanoparticles DOX@MSN@CuO_2_ (**Figure 7**) (78). DOX@MSN@CuO_2_ was capable of decomposing into Cu^2+^ and exogenous H_2_O_2_, while DOX was released from the weakly acidic TME. subsequently, Cu^2+^ reacted with the high level of GSH in TME, which induced GSH depletion and Cu^2+^ was reduced to Cu^+^. Cu^+^ and exogenous H_2_O_2_ produce toxic hydroxyl groups *via* the Fenton reaction, leading to apoptosis of tumor cells and thus enhancing CDT.

#### 3.7.2 pH/ROS

ROS is intimately interrelated with the progression of tumors. Over-accumulated ROS might induce apoptosis of cancer cells through oxidative stress. As such increasing ROS level in TME is a prospective remedy to destroy tumor cells. Exploring ROS and pH dual-responsive metal nanoparticles is currently the hottest spot in the field of tumor immunotherapy, which can initiate specialized release of therapeutic drugs in tumors through the stimulation of microenvironment inside tumors, so as to elevate anti-tumor efficacy and decline drug adverse effects. Chen and his colleagues have developed pH/ROS-responsive nanoplatform (Fe^2+^@UCM-BBD), which contains DOX, Fe^2+^ and Ce6 (80). Fe^2+^@UCM-BBD releases adriamycin in the acidic tumor microenvironment, which can induce tumor apoptosis through DNA damage. In a further, DOX also enhances the production of H_2_O_2_, which was able to produce enough material for CDT. The nanoparticles then responsively release Fe^2+^ for CDT based on high level of ROS in the TME. Generally, Fe^2+^@UCM-BBD presented great potential for clinical oncology therapy by demonstrating targeted release of therapeutic drug capability and superior tumor suppression through combination therapy.

#### 3.7.3 pH/Hypoxia

It has been demonstrated that aerobic glycolysis and excessive accumulation of lactate are both connected with the establishment of a tumor immunosuppressive microenvironment. M2 macrophages, which are known as anti-inflammatory immune cells, facilitate tumor growth and angiogenesis.

Weakly acidic TME can lead to the infiltration of M2 macrophages in tumors and foster tumor progression. Parallel to this, polarized M2 macrophages has ability to induce monocytes to express immunosuppressive transmembrane proteins, thereby blocking the adaptive immune response. Under such circumstances, synthetic multi-responsive metal nanoparticles may potentially revert the tumor immunosuppressive state and accomplish inhibition of tumor progression by alleviating the hypoxia and pH of the tumor microenvironment. Zhang et al. formed a nanocomplex (PALF) by cross-linking polyphenol derivatives with ferrous ions and then introducing lactate oxidase and the mitochondrial respiration inhibitor atovaquone (81). In this nanocomplex, lactate oxidase promoted lactate catabolism, polyphenol derivatives induced ROS accumulation, and atovaquone led to mitochondrial dysfunction, which together reduced oxygen consumption. PALF exhibited high efficiency immunostimulatory properties by modulating tumor microenvironment hypoxia and pH, promoting the release of inflammatory factors in tumor tissues, reduced polarization of M2 macrophages, and increased infiltration of activated T cells. PALF reversed the tumor immunosuppressive state and successfully inhibited tumor proliferation and metastasis, which has probably precluded the application of multi-responsive metal nanoparticles for tumor immunotherapy. Although multi-responsive metal nanoparticles could enhance the controlled release of drugs and improve therapeutic efficiency, the complexity of the design and fabrication processes may affect the potential for translation into clinical applications. It is therefore essential to improve and simplify the preparation of nanomedicines to boost the development of cancer immunotherapy.

## 4 Discussion

Despite the considerable progression of tumor immunotherapy, the availability of immunosuppressive TME has hindered its clinical application. The application of targeted drugs has the potential to enhance the effectiveness of antitumor immunotherapy while decreasing side effects., However, the distinctive characteristic of TME provides an excellent possibility to exploit them as targets for cancer immunotherapy. Metal nanoparticles have been intensively investigated as one of the promising candidates for novel targeted antitumor therapies due to their superior flexible physical and chemical properties. Studies so far have shown that metal nanoparticles provide several advantages, including ease of synthesis, low fabrication cost and flexibility in controlling the form and size of nanoparticles.

Many metals and metallic elements could be formed into nanostructures, which can be engineered and manufactured by diverse synthetic and functionalization methods. It is reported that metal nanoparticles have ability to be surface functionalization with polymers, surfactants, drugs, antibodies, peptides, and oligonucleotides to enhance their ability to targeted delivery of drugs and therapeutic applications. The increasing number of studies related to metal nanoparticles in oncology therapy is due to their inherent characteristics such as unique high drug payload, ease of functionalization with biomolecules, low toxicity, optical properties, high surface area, and radiosensitizer properties. However, metal nanoparticles likewise have a special drawback regarding high aggregation, which due to the large weight, skin color changes, clearance problems and high price. Therefore, further studies should to be explored for evaluating the pharmacokinetic and toxicity properties of the metal nanoparticles before they could be used in clinical applications.

TME-responsive metal nanoparticles have shown great promise in addressing the challenges associated with cancer immunotherapy. TME-responsive metal nanoparticles focus on unique chemical features in TME such as pH, GSH, ROS, hypoxia, enzymes and ATP. TME-responsive metal nanoparticles are also capable of integrating immunotherapy with multiple therapeutic regimens, presenting the tremendous merit of enhancing the efficiency of tumor immunotherapy with reduced adverse effects through the controllable release of drugs and tumor site-specific accumulation. In spite of the widespread progress in research related to the use of metal nanoparticles for tumor immunotherapy, there are still some application constraints and challenges. The most pivotal challenges that require consideration in future research are targeting and toxicity

The biosafety of metal nanoparticles is a basic requirement for its medical application and clinical translation. Future studies should explore systematically the biosafety of metal nanoparticles in combination with immunotherapeutic agents so as to identify the immune system dysfunction and host tissue damage that may result from long-term application. In addition, the results of mouse models typically do not reflect the true effect on human tumour due to the high heterogeneity of human tumor, which increases the complexity of clinical translation of metal nanoparticles. The integration of metal nanoparticles with immunotherapy is in a thriving stage of evolution.

However, TME-responsive metal nanoparticles for immunotherapy have not been used in clinical trials yet. The fabrication of TME-responsive nanodrugs has high complexity, which may be the main constraint that makes it difficult to produce it in a large scale and stable manner. Consequently, it is also quite significant to reduce the fabrication complexity of current metal nanoparticles for their clinical translation. At the same time, it is essential to have a range of industry standards related to the manufacture and use of metal nanoparticles before using it in clinical trials. Specific precautions include controlling the reproducibility and stability of nanoparticle preparation techniques, determining the level of drug accumulation at the target site, and toxicological hazards. The regulatory agencies are required to play an influential role in setting standards for the clinical use of metal nanoparticles and in the assessment of efficiency.

## 5 Conclusion

While there are many problems that remain to be solved, metal nanoparticles have introduced innovative approaches to the treatment of tumor immunotherapy, and their application in biology warrants further investigation and development. TME-responsive metallic nanoparticles exhibit the advantage of controlled drug delivery and accumulation, which may reduce the toxicity of immunotherapy and immune-related adverse events. The combination of metal nanoparticles with immunotherapy allows not only to destroy tumors, but also to activate a systemic anti-tumor immune response by initiating the release of tumor antigens and intracellular danger signals, resulting in the suppression of tumor metastasis and recurrence. In spite of the significant advances currently being made in these technologies, the majority of nanoparticles described in this review remain in the preliminary design and optimization stages and in human patients have not yet been tried and tested. Among the greatest existing challenges in the field of TME-targeted metal nanoparticle antitumor immunotherapy is the translation to the clinic, which will eventually define the viability and effectiveness of the approach. Undoubtedly, immunotherapy in combination with TME-responsive metal nanoparticles has tremendous clinical potential and will become an invaluable clinical tool in the combat against cancer.

## Author contributions

YZ conceived and designed the current study. RY and LC search and summarize the data. YW, LZ and YY analyzed the data. RY, LC and YY drafted the manuscript. All authors contributed to the article and approved the submitted version.
